# Role of OCT in Assessing Vasa Vasorum in Chronic Coronary Syndrome: Insights from Long-Term Follow-Up

**DOI:** 10.3390/jcm14051560

**Published:** 2025-02-26

**Authors:** Piotr Baruś, Karolina Bartkiewicz, Piotr Pęczek, Anna Libera, Piotr Dunaj, Szymon Jonik, Janusz Kochman, Marcin Grabowski, Mariusz Tomaniak

**Affiliations:** First Department and Clinic of Cardiology, Medical University of Warsaw, 02-097 Warszawa, Poland; piotr.barus@wum.edu.pl (P.B.); karolina.bartkiewicz2000@gmail.com (K.B.); dunajpiotr98@gmail.com (P.D.); szymonjonik.wum@gmail.com (S.J.);

**Keywords:** vasa vasorum, chronic coronary syndrome, optical coherence tomography

## Abstract

**Background/Objectives**: The aim of this study was to analyze the presence of vasa vasorum in optical coherence tomography (OCT) among patients undergoing coronary angiogram for chronic coronary syndrome with intermediate-grade coronary stenoses in relation to long-term follow-up. **Methods**: This prospective, observational, single-center study enrolled patients with chronic coronary syndrome and intermediate-grade coronary stenosis. OCT was used to assess the presence of vasa vasorum, type of plaque, mean lumen area, fibrous cap thickness (FCT), and minimal lumen diameter. Patients were divided into two groups based on the presence of vasa vasorum. **Results**: Overall, 97 patients were enrolled, of whom 82.5% were male. Lesions with vasa vasorum were found in 76 patients. Comorbidities such as diabetes mellitus, hypertension, dyslipidemia, and chronic kidney disease did not differ significantly between groups. Among patients with vasa vasorum, there were higher serum creatinine levels (1.03 ± 0.24 vs. 0.87 ± 0.22, *p* = 0.009). OCT showed that minimal lumen diameter differed between groups (2.26 ± 0.38 mm vs. 2.57 ± 0.57 mm *p* = 0.026) for the vasa vasorum group and no vasa vasorum, respectively, however minimal lumen area was similar in both groups (3.88 ± 1.76 mm^2^ vs. 4.01 ± 2.00 mm^2^, *p* = 0.731, for vasa vasorum and no vasa vasorum, respectively). Furthermore, the presence of vasa vasorum seemed to have no significant correlation with cardiovascular events in the 2-year, 5-year, and 10-year follow-up. **Conclusions**: The presence of lesions with vasa vasorum was not shown to be linked to any unfavorable patients’ outcomes. Among men, coronary atherosclerotic plaques were more likely to contain OCT-visualized vasa vasorum.

## 1. Introduction

**Figure 1:**
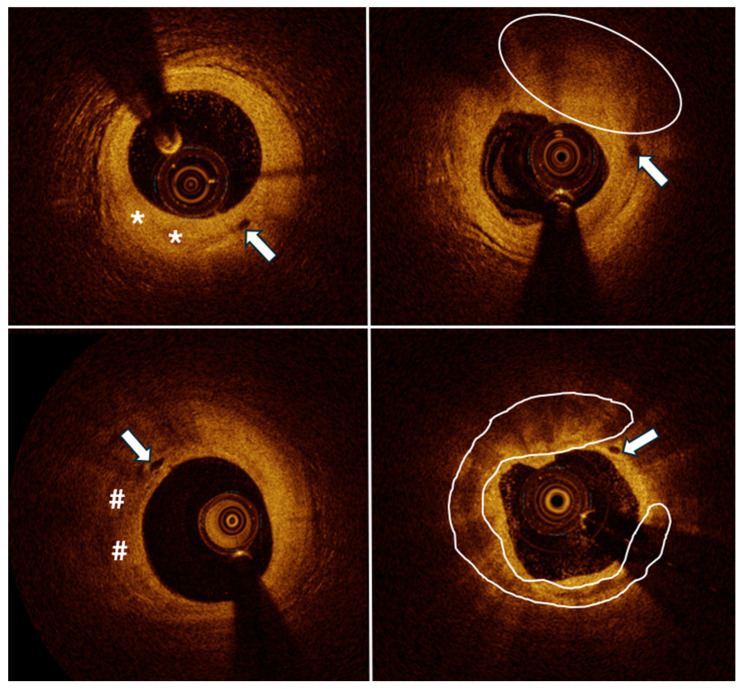
Examples of vasa vasorum (marked with arrows). *—fibrous lesion, #—lipidic lesion, oval marking—mixed plaque type, and c shape marking—calcific plaque.

Optical coherence tomography (OCT) is an intravascular modality offering a comprehensive visualization of the vessel wall. It allows for differentiation of plaque types, identification of high-risk plaques, and diagnosis of plaque rupture or erosion, which lead to appropriate treatment [1,2]. The image is created based on the analysis of infrared light reflected from the vessel wall depending on the return time to the device emitting the light beam. It is believed that it is currently possible to obtain reliable imaging in the range of up to 2 mm of blood vessel wall thickness. In turn, modern OCT technology allows us to generate images with axial resolution of 12–18 µm and transverse resolution of 20–90 µm [3]. Its value is widely acknowledged for percutaneous coronary intervention (PCI) guidance. This modality is present in the recent guidelines for optimal stent implantation [4,5]. OCT constitutes an effective diagnostic method for imaging the internal structure of coronary vessels, including detection of vasa vasorum (VV) (Figure 1). VV are characterized by a diameter of 50–300 µm, playing an important role in delivering blood to the arterial wall [6]. However, adventitial VV and the arising neovessels can lead to a hemorrhage within the plaque [7]. The formation of lesions within coronary arteries results from the accumulation of lipids and inflammatory cells, causing inflammation [8,9]. Moreover, evidence suggests that the atherosclerotic process begins in the adventitia, where VV are present, and eventually reaches the media and intima [8,9]. Taking this evidence into account, studies have been performed to examine the association of VV with characteristics of plaque vulnerability and have proven the association [10,11,12]. While some studies concentrated on VV ex vivo and some in vivo, to our knowledge, the relationship between VV visualized by OCT and patients’ follow-up has not been evaluated. The aim of this study was to assess the connection between the presence of VV among patients undergoing coronary angiogram (CAG) for chronic coronary syndrome (CCS) with intermediate-grade coronary lesions in angiography and long-term follow-up.

## 2. Materials and Methods

This was a single-center, prospective, observational study consecutively enrolling patients with CCS and intermediate-grade coronary stenosis evaluated by OCT. The inclusion criteria have been previously published [13,14,15] and comprised the following: chronic coronary syndrome, severity of anginal pain of 2–3 in the Canadian Cardiovascular Society classification or positive ischemia test (single photon emission tomography or exercise test), age of 18 years or more, presence of intermediate-grade coronary stenoses [16], and OCT examination. Exclusion criteria involved the following: ostial left main disease, ostial right coronary lesion, history of coronary artery bypass grafting, hemodynamic instability, acute or chronic renal insufficiency (serum creatinine level > 1.5 mmol/L), and pregnancy. The research protocol received approval from the local research ethics committee and adhered to the principles outlined in the Declaration of Helsinki. Written informed consent was obtained from all participating patients.

Acquisition of OCT images was performed with a frequency domain OCT imaging system (Abbott, C7XR Dragonfly TM, LighLab Imaging Inc., Westford, MA, USA) with the use of non-occlusive technique. Pullbacks were analyzed at 0.2 mm intervals by analysts blinded to patients’ clinical presentation and comorbidities as well as angiographic data.

The analysis of OCT images was guided by the latest expert consensuses [17,18,19,20]. Assessment of the reference lumen area entailed examining the largest lumen either preceding or following a stenosis (within 10 mm of the stenosis). Morphometric analysis of plaques was performed at the location of the minimal lumen area (MLA) across a minimum of three consecutive frames. Plaques were classified as calcified, fibrous, lipid-rich, or mixed. Calcified plaques exhibit a signal-poor, heterogeneous region with a sharply delineated border, whereas a fibrous plaque is characterized by high backscattering and a relatively homogenous signal [17,18,19,20]. A plaque was deemed lipid-rich when it presented an irregular signal-poor region with diffused borders [18,21]. Fibrous cap thickness (FCT) denotes the distance from the inner edge of the lipid or calcium pool to the border of the vessel lumen. FCT measurements were conducted at 0.2 mm intervals along the plaque. Following this, three measurements were obtained at the thinnest segment of the plaque within each cross-section, and the resulting average value was utilized for the final analyses [21]. A plaque exhibiting a fibrous cap thickness (FCT) of less than 65 µm was categorized as a thin-cap fibroatheroma (TCFA) [20].

The primary outcome of the study was overall mortality. The secondary outcome included major adverse cardiovascular events (MACE), defined as cardiac death, myocardial infarction, stroke, unplanned PCI or hospitalization due to heart failure.

Statistical analyses were carried out using SPSS version 28.0 (IBM Corp, Armonk, NY, USA). The distribution of collected data was evaluated using the Kolmogorov–Smirnov test. Normally distributed data were presented as means and standard deviations (SD), while non-parametric data were described using median values and the interquartile range (IQR) between the 25th and 75th percentiles. Categorical data were expressed as percentages within their respective groups. Group comparisons were performed using Student’s *t*-test for normally distributed data and the Mann–Whitney U test for non-normally distributed continuous variables. Categorical variables were analyzed using the chi-square test with Yates’s correction for continuity when applicable. Statistical significance was determined at a two-tailed *p*-value of less than 0.05. Survival and MACE-free survival during follow-up were assessed using the log-rank test and illustrated using Kaplan-Meier curves.

## 3. Results

A total of 97 patients (114 lesions) were analyzed. Overall, 82.5% (n = 80) of enrolled patients were male. Of the coexisting comorbidities, hypertension and dyslipidemia were most prevalent, 83.5% (n = 81) and 62.9% (n = 61), respectively. Individuals with diabetes constituted 34.0% (n = 33) of the participants and 30.9% (n = 30) had previously suffered at least one myocardial infarction (Table 1).

There were 76 patients with at least one coronary lesion with VV present, examples of which are in Figure 1. Patients in this group had higher serum creatinine levels (1.03 ± 0.24 vs. 0.87 ± 0.22, *p* = 0.009), lower eGFR (75.20 ± 21.00 vs. 86.08 ± 21.33, *p* = 0.043) and among men, coronary atherosclerotic plaques were more likely to contain OCT-visualized vasa vasorum than to be devoid of it (89.5% vs. 57.1%, *p* = 0.024) (Table 1).

OCT analysis did not reveal any differences in plaque morphology, nor in the presence of TCFA, between the groups. There was a significant difference in minimal lumen diameter, 2.26 ± 0.38 mm vs. 2.57 ± 0.57 mm (*p* = 0.026) for the VV group and no VV group, respectively, however mean lumen area was similar (3.88 ± 1.76 mm^2^ vs. 4.01 ± 2.00 mm^2^, *p* = 0.731, VV group vs. no VV group, respectively). Moreover, cap thickness over calcium in the distal part of the lesion was smaller (0.07 ± 0.06 mm vs. 0.18 ± 0.09 mm, *p* = 0.028) (Table 2).

**Figure 2:**
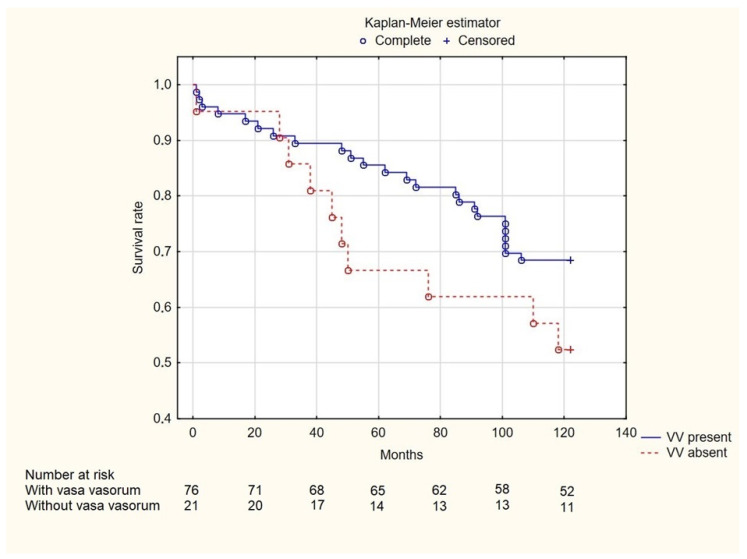
Kaplan-Meier overall survival curves of patients categorized according to presence of coronary vasa vasorum. VV—vasa vasorum.

**Figure 3:**
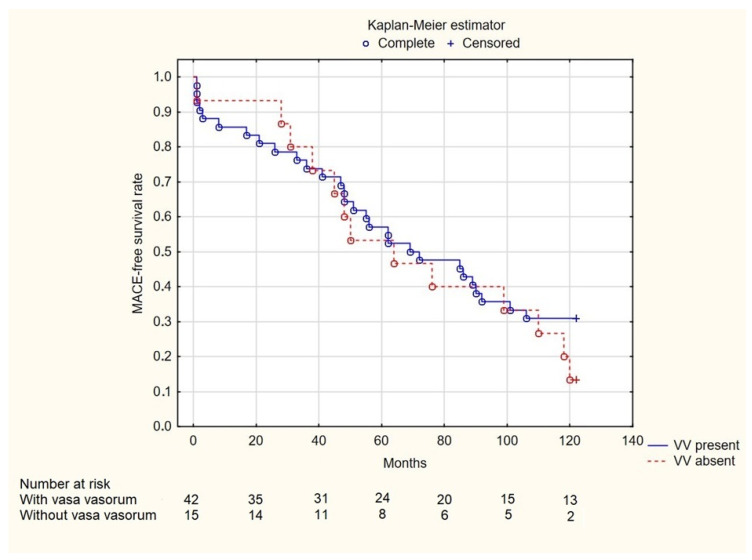
Kaplan-Meier MACE-free survival curves of patients categorized according to presence of coronary vasa vasorum. VV—vasa vasorum.

Information about time of death after enrollment was not available for nine patients with VV present and one patient with VV absent. Among the remaining patients, no significant differences were found in the 2-year (7.46% vs. 5%, *p* = 1.000), 5-year (14.93% vs. 35%, *p* = 0.096), and 10-year (31.5% vs. 47.6%, *p* = 0.158) mortality rates (Figure 2). Both groups were characterized by a similar occurrence of MACE (74% vs. 89.50%, *p* = 0.455) (Figure 3).

## 4. Discussion

This study sought to evaluate the influence of in vivo coronary VV on mortality rates. OCT was used to visualize in detail the plaques’ morphology and arterial wall, as well as to detect VV. From a structural viewpoint, there are two types of VV. First-order VV run longitudinally along the adventitial layer. Second-order VV can originate from the first-order VV or from coronary artery branches and run circumferentially to the lumen [22]. OCT is a relatively new imaging method with a plethora of studies still evaluating its uses across medical specialties. Prior to its invention, micro-computerized tomography (m-CT) was the only method applied to the visualization of VV. In a recent study, it has been proven that OCT is the most reliable way of analyzing first-order VV with m-CT permitting random error, which validates the accuracy of our findings [23]. Interobserver variability was introduced for evaluation of the OCT pullback videos. Different researchers calculated visible VV while the results were rechecked by an independent observer, limiting researcher bias.

There were no statistically significant differences in survival between patients with and without vasa vasorum in atherosclerotic changes in coronary arteries. This was true for the analyzed 2-, 5-, and 10-year time intervals. Similarly, there were no statistically significant differences in the occurrence of MACE based on the presence of coronary vasa vasorum. The reason for such observations could be the insufficiently representative group of patients analyzed by us. In addition, imaging of coronary arteries using OCT carries the risk of missing the presence of VV in atherosclerotic plaques. This may be due to the fact that VV are too deeply located or have too small a diameter. In such a situation, the depth of penetration of the vessel wall or the insufficient resolution of OCT technology could give such observations [3]. An extremely promising method of imaging heart microstructures is the developed m-CT method. It allows obtaining images with a resolution of even several micrometers. These are parameters comparable to the microscopic imaging of histopathological materials. However, m-CT allows in vivo assessment of the structure of, among others, atherosclerotic plaques [24]. This is an innovative method, and the possible visualization of VV plaques would significantly develop the understanding of their role in the course of coronary diseases.

The analysis of the structure of atherosclerotic plaques in coronary arteries is an important element of research aimed at improving the prognosis of patients with coronary artery disease. Atherosclerosis is an incurable disease [25,26]. However, implementing appropriate diagnostic and therapeutic procedures at an early stage of the disease may halt its progression. Intravascular imaging of changes in the walls of blood vessels allows for the detection of vulnerable plaques, which threaten to rupture and cause acute coronary syndrome [27]. Therefore, research on the influence of the presence of coronary VV in lesions is important to determine their role in the evolution of plaques. For example, Alfonso et al. [28] demonstrated an association between the plaque volume progression in heart transplant recipients (median of 2.1 years after heart transplant) and the presence of larger amounts of VV, suggesting that the presence of VV is linked to coronary artery disease progression. However, there are studies suggesting the possibility of stopping the progression of atherosclerotic plaques or even causing their regression using appropriate therapies that lower blood lipid levels and have anti-inflammatory effects [27]. Bhindi et al. [29], in their meta-analysis of 17 prospective studies evaluating the effect of dyslipidemia therapies on the occurrence of MACE, observed a positive effect of treatment on patient prognosis. A decrease of just 1% in the atheroma volume (PAV) was associated with a 20% reduction in the odds of MACE [29]. However, the high dynamics of the structure and intercellular interactions within the atherosclerotic plaque require further analysis, including of the role of VV. All this is aimed at preventing acute coronary syndromes and patient death [27].

Another interesting factor potentially related to VV is spontaneous coronary artery dissection (SCAD). A study by Jackson et al. [30] sought to determine the impact of the presence of VV and the mechanism of false lumen formation, as well as the type—fenestrated or non-fenestrated—on SCAD. It was shown that patients presenting with SCAD did not differ significantly compared to the control group in terms of VV density [30]. On the other hand, a study by Kwon et al. [31] presented different results, showing that patients with a history of SCAD present with a higher density of VV in nonculprit segments, adjacent to the SCAD region. Moreover, it has been reported that intraplaque neovessels may be associated with coronary spasm, however this has been described as a single case presentation and not in a study with a sufficient patient population [32]. In our study, we have only enrolled patients with chronic coronary syndrome and intermediate-grade stenoses, therefore we do not have available data to assess our findings in the setting of SCAD or vasospastic angina.

The results of the coronary artery examinations obtained by angiography and OCT that we analyzed show that statistically significantly higher values of mean lumen diameter (mm) and cap thickness over calcium at the distal part of the lesion (mm) were demonstrated in patients without coronary vasa vasorum. They were 2.57 ± 0.57 mm and 0.18 ± 0.09 mm, respectively. The differences in the remaining parameters depending on the presence of coronary vasa vasorum were not statistically significant. However, it was noted that in percentage terms, atherosclerotic lesions, both those containing and those not containing vasa vasorum, occurred most frequently in the LAD (approximately 60% of lesions were present here). On the other hand, atherosclerotic lesions occurred least frequently in the LM. Approximately 4% of atherosclerotic lesions contained vasa vasorum, and no lesions without their presence were found in LM. Moreover, in percentage terms, calcified plaque was the most common (approximately 35% of lesions with vasa vasorum and 43% of lesions without vasa vasorum), and lipid-rich plaque was the least common (approximately 16% of lesions with vasa vasorum and approximately 3% of lesions without vasa vasorum). Overall, 16% of lesions with vasa vasorum were also of mixed structure. The results of this study differ from those published by other researchers. This may be due to the relatively small and poorly diverse group of patients we had. Large trials are needed to examine this matter in more detail. In the research study conducted by Taruya et al. [10], which included 53 participants, the proximal left anterior descending was analyzed. This study also showed that VV are more prevalent in the fibrous type of lesions [10]. The primary difference between the mentioned study and ours is that we have analyzed all coronary vessels and not only the left anterior descending, leaving a potential explanation for the different results. Moreover, we have analyzed the follow-up of patients and Taruya et al. [10] did not, therefore it is impossible to state whether the increased plaque vulnerability characteristics as detected by OCT had a clinical influence. On the contrary, a study by Amano et al. [22] evaluated the slow flow during PCI in relation to the presence of longitudinal running VV within the plaque (defined as neovessels running from the adventitia to the plaque). In total, 71 lesions were analyzed by OCT, of which 33 lesions (47%) had internal running VV. Lesions with VV present were more frequently lipidic, with a higher prevalence of intimal laceration and plaque rapture, as well as TCFA. Moreover, among those lesions, after stent implantation slow flow was present [22].

A statistically significant result of this study is that serum creatinine levels were within the normal range for adults, both in the presence and absence of coronary vasa vasorum among the analyzed patients. Also, eGFR values differed statistically significantly in the mentioned groups of patients, reaching values of 75.2 ± 21 mL/min/1.73 m^2^ and 86.1 ± 21.3 mL/min/1.73 m^2^, respectively, in the presence and absence of coronary vasa vasorum. As a rule, the eGFR value should be at least 90 mL/min/1.73 m^2^ in healthy individuals. However, factors such as age and gender modify this value. In addition, the determination of impaired renal excretory function requires the assessment of other parameters of the patient’s clinical condition (laboratory—albuminuria, urine sediment assessment, and renal tubule function—imaging or histopathological) which occur over a sufficiently long period of time (minimum 3 months in the case of chronic kidney disease) [33]. Based on the data we have, we are unable to formulate statistically significant, detailed conclusions on the relationship between eGFR, serum creatinine levels, and the presence of coronary vasa vasorum in patients with a specifically defined clinical condition. Chronic renal failure increases cardiovascular risk in patients [34]. Therefore, precise determination of the impact of renal disease on the stability of atherosclerotic plaques seems clinically crucial.

We did not observe that the presence of specific diseases among the analyzed patients had an impact on the presence/absence of VV in atherosclerotic lesions. No statistically significant correlations were found between the presence/absence of coronary vasa vasorum, specific nosological entities (diabetes, hypertension, dyslipidemia, chronic kidney disease, chronic heart failure, previous myocardial infarction, transient cerebral ischemic attacks (TIA), previous stroke, smoking), age, and previous PCI or CABG procedures among the patients analyzed by us.

It is clearly demonstrated that studies concerning the presence of VV produce contrasting results. To our knowledge, our study is the first to evaluate VV in a long-term follow-up of chronic coronary syndrome with intermediate coronary stenosis. Our observations shed a new light on coronary VV and their clinical impact. Moreover, the differences among the groups in our study, although statistically insignificant in the current form, should perhaps be evaluated in larger populations, with emphasis on long-term observation. Additionally, concentration on vessel-specific outcomes may further deepen the knowledge.

This study has some limitations, which concern both group of patients that we analyzed, and the coronary artery imaging method used, i.e., OCT.

We managed to include relatively few patients in this study, i.e., 97 participants. They were mostly men (80 patients), and only 17 were women. All were Caucasian. Participants in the study were patients of one hospital, which could have caused selection bias.

Intravascular OCT has limitations of a physical nature, which result from the principle of tissue imaging. Therefore, the OCT technology used has limitations in terms of the possible depth of tissue penetration and the resolution of the images obtained. It is difficult to determine the presence of VV that are separated from the OCT catheter by tissue (for example, calcified atherosclerotic plaque), which reflects a significant part of the emitted infrared light [3]. Due to the limited penetration depth of OCT [35], it can also be argued that this method should only be used in the early stages of vasculopathy and not advanced lesions [28]. Better penetration of the vessel wall is achieved with the help of ultrasound waves in intravascular ultrasound (IVUS) imaging. With IVUS, tissues can be imaged to a depth of about 4–8 mm. However, the images obtained have an axial and lateral resolution of 150–200 µm and 150–300 µm, respectively [3]. These are significantly worse values than those currently available with OCT. Due to the small diameter of VV (50–300 µm), their imaging using IVUS is very difficult. A reliable imaging method is the previously mentioned m-CT, which is currently being developed. However, more studies are currently needed to confirm the possibility of using this method in detecting VV in atherosclerotic plaques.

In order to acquire a good quality of OCT images, it is necessary to inject contrast into the vessel. Therefore, according to Lame’s law, there is a possibility that injection of the contrast creates a pressure gradient so big it results in the compression of some VV, limiting their final number [10]. The results of the analysis of various imaging studies may be to some extent burdened with someone’s subjective assessment. Therefore, the identification of the presence or absence of VV within the lesions in the OCT imaging results could have been influenced by the researcher’s previous experience.

All this makes it impossible to formulate reliable generalizations based on the observations we have obtained. In order to obtain statistically significant results, it is necessary to conduct multicenter studies of a larger group of patients with different individual characteristics.

## 5. Conclusions

The results of this study indicate a statistically significantly higher incidence of coronary atherosclerotic plaques containing vasa vasorum compared to the incidence of lesions without vasa vasorum in the analyzed group of male patients. Demonstrating a similar, statistically significant relationship in female patients, as well as differences between men and women, was not possible in our study. This is due to the disproportionately larger number of men compared to women in the group of patients analyzed by us. Therefore, conducting a reliable direct comparative analysis between the sexes is not possible based on the data we currently have. Moreover, the presence of lesions with vasa vasorum was not shown to be linked to any unfavorable outcomes among all the study participants that we analyzed.

## Figures and Tables

**Table 1:** Baseline characteristics of patients categorized according to presence of coronary vasa vasorum. eGFR—estimated glomerular filtration rate, PCI—percutaneous coronary intervention, CABG—coronary artery bypass grafting, MI—myocardial infarction, and TIA—transient ischemic attack. Data are presented as count (%), mean and standard deviation (±), or median and interquartile range (IQR).

Feature	Present Coronary Vasa Vasorum (n = 76)	Absence of Coronary Vasa Vasorum (n = 21)	*p*-Value
Age (years)	64.79 ± 9.18	64.57 ± 9.68	0.924
Male	68 (89.5%)	12 (57.1%)	**0.024**
Diabetes mellitus	25 (32.9%)	8 (38.1%)	0.853
Hypertension	61 (80.3%)	20 (95.2%)	0.181
Dyslipidemia	50 (65.8%)	11 (52.4%)	0.384
Chronic kidney disease	10 (13.2%)	1 (4.8%)	0.447
Serum creatinine (mg/dL)	1.03 ± 0.24	0.87 ± 0.22	**0.009**
eGFR	75.2 ± 21	86.1 ± 21.3	**0.043**
Chronic heart failure	12 (15.8%)	4 (19.1%)	0.744
Previous PCI	55 (72.4%)	16 (76.2%)	0.943
Previous CABG	3 (4.0%)	1 (4.1%)	1.000
Previous MI	23 (88.5%)	7 (87.5%)	0.984
TIA/stroke	1 (1.3%)	1 (5.0%)	0.375
Current smoker	12 (16.0%)	4 (19.1%)	0.746

**Table 2:** Angiographic characteristics and optical coherence tomography measurements. LM—left main, LAD—left anterior descending, Cx—circumflex artery, RCA—right coronary artery, CAG—coronary angiography, TCFA—thin-cap fibroatheroma, and FCT—fibrous cap thickness. Data are presented as count (%), mean and standard deviation (±), or median and interquartile range (IQR).

Feature	Present Coronary Vasa Vasorum (n = 84)	Absence of Coronary Vasa Vasorum (n = 30)	*p*-Value
LM	3 (3.6%)	0 (0.0%)	0.565
LAD	50 (59.5%)	18 (60.0%)	0.864
Cx	7 (8.3%)	5 16.7%)	0.352
RCA	19 (22.6%)	6 (20.0%)	0.968
Lesion length in CAG (mm)	14.9 ± 8.3	14.7 ± 6.5	0.760
Diameter stenosis in CAG (%)	57.6 ± 13.8	60.7 ± 15.3	0.311
Mean lumen area (mm^2^)	3.88 ± 1.76	4.01 ± 2.00	0.731
Mean lumen diameter (mm)	2.26 ± 0.38	2.57 ± 0.57	**0.026**
Calcified plaque	29 (34.9%)	13 (43.3%)	0.552
Fibrous plaque	28 (33.7%)	10 (33.3%)	0.853
Lipid-rich plaque	13 (15.7%)	1 (3.3%)	0.108
Mixed plaque	13 (15.7%)	6 (20.0%)	0.795
TCFA	14 (16.9%)	5 (16.7%)	0.795
Mean FCT (mm)	0.14 ± 0.05	0.16 ± 0.06	0.309
Minimal FCT (mm)	0.13 ± 0.05	0.16 ± 0.06	0.131
Mean angle of the calcium (°)	115.1 ± 63.8	109.7 ± 63.2	0.670
Maximal angle of the calcium (°)	132.8 ± 77.8	120.8 ± 80.5	0.383
Mean cap thickness over calcium (mm)	0.11 ± 0.07	0.12 ± 0.08	0.630
Cap thickness over calcium at the distal part of the lesion (mm)	0.07 ± 0.06	0.18 ± 0.09	**0.028**

## Data Availability

The data presented in this study are available on request from the corresponding author due to legal limitations.
